# Direct Synthesis of ESBO Derivatives-^18^O Labelled with Dioxirane

**DOI:** 10.1155/2013/212156

**Published:** 2013-09-12

**Authors:** Stefano La Tegola, Cosimo Annese, Michele Suman, Immacolata Tommasi, Caterina Fusco, Lucia D'Accolti

**Affiliations:** ^1^Chemist at Agenzia delle Dogane e dei Monopoli, Italy; ^2^Department of Chemistry, Università degli Studi di Bari Aldo Moro, Via Orabona 4, 70126 Bari, Italy; ^3^CNR-Istituto di Chimica dei Composti Organometallici (ICCOM), Sezione di Bari, Via Orabona 4, 70126 Bari, Italy; ^4^Barilla G. R. F.lli SpA, R&D Central Research Labs, Via Mantova 166, 43100 Parma, Italy

## Abstract

This work addresses a new approach developed in our laboratory, consisting in the application of isolated dimethyldioxirane (DDO, **1a**) labelled with ^18^O for synthesis of epoxidized glyceryl linoleate (Gly-LLL, **2**). We expect that this work could contribute in improving analytical methods for the determination of epoxidized soybean oil (ESBO) in complex food matrices by adopting an ^18^O-labelled-epoxidized triacylglycerol as an internal standard.

## 1. Introduction 

The packaging has an essential rule in the food manufacture: it increases the life of food, while guarantees protection from physical and microbiological contaminations [1]. 

**Figure 1:**
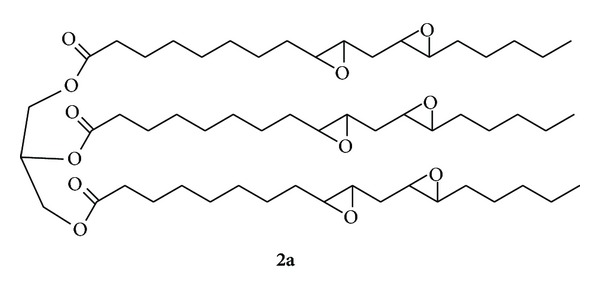
Structure of (Gly-LLL-ox **2a**): one of chemical species in ESBO (from linoleic in sn-1, sn-2, and sn-3).

In order to improve the packaging materials performances, in recent decades the use of different types of additives has been significant development (plasticizers, stabilizers, etc.) [2–4], like vegetable oils, that represent an interesting renewable source for the production of useful chemicals and new materials [5–8]. In particular, the use of epoxidized soybean oil (ESBO; see Figure 1) has increased in recent years as a valid alternative to phthalates, since it shows relatively good compatibility with PVC and low toxicity to humans [9]. In this respect ESBO is listed as an authorized substance according to food packaging materials legislation (European Commission 2002).

However, a known potential is the migration of ESBO from the packaging into the food, especially oily foods and fats. This risk in food contamination was investigated by the European Food Safety Authority (EFSA), which set a specific migration limit for ESBO in baby foods [10]. Several analytical methods have been reported for the analysis of ESBO in PVC gaskets and food, although they are very time-consuming and not so user-friendly, especially due to low concentrations of migrants and the complexity of the food matrix [10, 11].

The aim of this work was to obtain a selective synthesis of exa-epoxy-three linoleate glyceryl-^18^O labelled (**2a**) in one or multiple positions. This target compound could be used as internal standard in quantitative ESBO analysis, in order to simplify and increase the accuracy of our previous LC-MS/MS method [10].

**Scheme 1:**
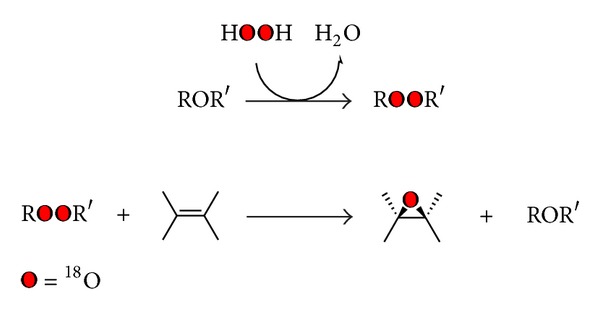


Despite the fact that the synthesis of ESBO [12] of epoxy esters of unsaturated fatty acids [13] could be obtained by different methods yielding conversion and selectivity higher than 90%, the preparation of its ^18^O labelled epoxide is quite difficult [14]. A possibility is to prepare the corresponding peroxide compound labelled R^18^O^18^OR′, generated from hydrogen peroxide-^18^O-^18^O (Scheme 1) [15].

The standard procedure now mentioned is very arduous and delicate if one takes into account the fact that hydrogen peroxide-^18^O-^18^O is synthesized by passing H_2_^18^O vapour through a tube discharge (V 2000) [16] or using 97% ^18^O labelled O_2_ gas [17].

**Figure 2:**
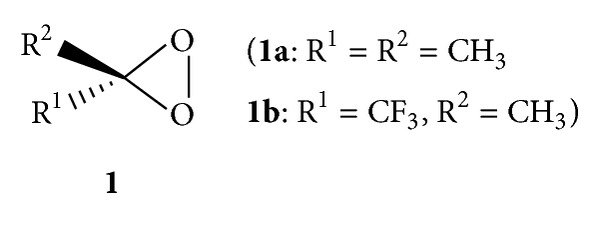
Dioxiranes commonly employed in the isolated form (in solution of the parent ketone).

In this context, we have shown that the dioxiranes R^1^R^2^CO_2_ (**1**) [18, 19] three-member ring strained peroxides, generated *in situ*, were able to transfer electrophilic oxygen to a variety of donor substrates (S:), yielding the corresponding oxidation products (SO) and the parent ketone (Figure 2).

Undoubtedly very attractive applications of these reagents are regio-, stereo-, and enantioselective epoxidations of alkenes. In fact simple [18] or supported ketones [20] and optical active ketones as precursors of chiral dioxiranes *in situ* [21, 22] could be used in organic synthesis.

**Scheme 2:**
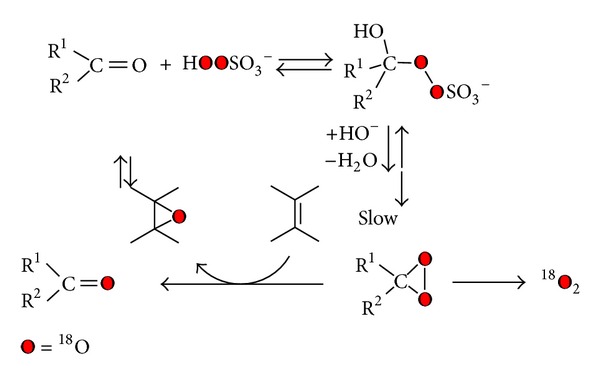


Further a possible alternative to obtain labelled epoxides consists of using DDO (**1a**) ^18^O labelled, generated *in situ*, from acetone and potassium monoperoxysulfate (KHSO_5_) K + -O-SO_2_-^18^O^18^OH (caroate) ^18^O-labelled—as reported in Scheme 2 [23].

This procedure requires a careful preparation, since the caroate is also able to give molecular oxygen, with loss of ^18^O_2_, as background reaction [19].

Until now the setup of appropriate techniques allowed the isolation of a few dioxirane volatile species, such as dimethyldioxirane (CH_3_)_2_CO_2_ (**1a**) [24] and the powerful methyl(trifluoromethyl)dioxirane (CF_3_)(CH_3_)CO_2_ (**1b**) [25]. These dioxiranes have been found to be a versatile epoxidizing reagent for alkenes of various kinds, either electron rich or electron poor [26] and allylic alcohols [27].

In continuation of our work on selective polyepoxidations [28, 29] we now report on the epoxidation of glyceryl-trilinoleate (**2**) using the isolated ^18^O DDO (**1a**). Our preliminary results are described herein. 

## 2. Materials and Methods

The GC analyses were run using a ZB-1 column (30 m × 0.25 *μ*m id); in most cases, 1,1,1,2-tetrachloro-2,2-difluoroethane (Freon A112) was employed as inert internal standard. The ^1^H NMR were recorded on a 500 MHz resonances and are referenced to residual isotopic impurity CHCl_3_ (7.26 ppm) of solvent CDCl_3_ and/or to TMS. The ^13^C NMR spectra (125.76 MHz) are referenced to the middle peak of CDCl_3_ solvent (77.0 ppm). MS experiments were performed using a Micromass M@LDITM–LR (Waters MS Technologies, Manchester, UK) TOF mass spectrometer equipped with a nitrogen UV laser (337 nm wavelength).

Commercial acetone and methylene chloride (AnalaR grade) were purified by standard methods, stored over 4 Å molecular sieves. H_2_O labelled with ^18^O (isotopic purity 95%), sulfuric acid (H_2_SO_4_), calcium chloride (CaCl_2_), magnesium sulfate (MgSO_4_), sodium hydroxide pellets (NaOH), sodium thiosulfate (Na_2_S_2_O_3_), methyl p-tolyl-sulfide, disodium salt of ethylenediaminetetraacetic acid (EDTA·Na_2_), and sodium bicarbonate (NaHCO_3_), commercial products at high purity degree, were used without further purification.

Curox triple salt 2KHSO_5_·KHSO_4_·K_2_SO_4_ (a gift by Peroxid-Chemie GmbH, Munich, Germany) was our source of potassium peroxymonosulfate (caroate); it was used as received for the synthesis of dioxiranes **1a**.

### 2.1. Preparation of ^18^O-DDO (**1a**)

Dioxiranes in solution of the parent ketones (i.e., acetone for **1a**) could be obtained by following a procedure already reported in detail [24, 30] starting with buffered (pH 7, NaHCO_3_) aqueous potassium peroxomonosulfate KHSO_5_ and the ketone. A 500 mL four-necked round-bottomed vessel is equipped with a mechanical stirrer, a splash-guard adapter connected to an air-cooled straight condenser, a solids addition funnel, a gas inlet tube extending into the reaction mixture, and a thermometer. The air condenser is connected laterally to the top entry of an efficient jacketed spiral condenser cooled at −70°C; the bottom exit of this condenser is fitted to a two-way fraction-collector adapter carrying two 50 mL receiving flasks and is also cooled at −70°C. In succession to the adapter, two cold traps are placed, the first is cooled at −70°C and the second at liquid nitrogen temperature. The main vessel is charged with 35 mL of bidistilled water, the mixture of ^18^O acetone (30 mL) and H_2_^18^O (6 mL) Na_2_EDTA_2_ (0.1 g), and NaHCO_3_ (22 g). Mechanical stirring is initiated, and the solid potassium peroxomonosulfate (0.189 mol, 66 g of corresponding to 2.86 mmol/g of Caroat triple salt iodometry) is quickly added during 1-2 minutes to the reaction vessel, while passing a gentle stream of Ar (or N_2_) gas through the mixture. Shortly after the addition is initiated, the end two-way adaptor is switched to insert the main collection flask and during 10 min ca. 25 mL of weak-yellow solution of **1a** in acetone is collected. This solution had a 0.083 M concentration in dioxirane (iodometry).

### 2.2. General Procedure for Oxidation of Methyl-p-tolyl Sulfide (**3**) with Dioxirane ^18^O-DDO (**1a**)

A solution of methyl-p-tolyl-sulfide (**3**) (9.1 mg, 0.066 mmol) in 5 mL of CH_2_Cl_2_ dry was prepared. To 0.3 mL of this standard solution kept at 0°C 0.01 mL of ^18^O-DDO (**1a**) solution 0.083 M in acetone (2 equivalents) was added. The reaction progress was monitored by GC analysis of microaliquots periodically withdrawn from the reaction flask. In each experiment, the values were measured at various reaction times; data from two or more independent runs were averaged. Upon reaction completion, the reaction mixture was characterized by a combination of spectroscopic techniques (^1^H, ^13^C NMR, IR, and MS) collecting data in agreement with the literature. The mass spectrum of the resulting labelled **3a** revealed two peaks at *m*/*z* (ri) 154 (M^+^, 100) and 156 (M^+^ + 2, 13.9), while for the same unlabelled **3a** *m*/*z* ****154 (M^+^, 100) and 156 (M^+^ + 2, 5.9). The percent of ^18^O was estimated from the 100 × {[(M+2/M)_labelled_ − (M+2/M)_unlabelled_]}.

### 2.3. Oxidation of Glyceryl-trilinoleate with ^18^O-DDO (**1a**)

To a solution of Gly-LLL (**2**) (0.213 g 0.24 mmoli), in 20 mL of CH_2_Cl_2_ dry, kept at 20°C, a solution ^18^O-DDO (**1a**) 0.083 M in ^18^O acetone of 21 mL (7.2 equiv.) was added. The reaction was monitored using TLC (silica gel hexane/ethyl ether 50 : 50). The precious labelled acetone was recovered by careful distillation of the crude reaction (18 mL, 20% labelled). Upon completion, pure ^18^O-LLL_ox_  (**2a**): was simply obtained by evaporation of the volatile solvents *in vacuum* and full characterized with MALDI-Tof MS ^1^H e {^1^H}  ^13^C-NMR.

^1^H-NMR (CDCl_3_, 500 MHz): *δ* 0.86 (m, 9H, CH_3_), 1.25–1.80 (m, 66H, CH_2_), 2.28 (s, 6H, CH_2_ in *α* al C=O), 2.90–3.08 (m, 12H, CH epox), 4.11–4.22 (m, 4H, CH_2_ glycerol), 5.22 (m, 1H, CH glycerol); {^1^H}  ^13^C-NMR (CDCl_3_, 125 MHz): *δ* 13.89, 22.47, 24.67, 24.70, 26.04, 26.14, 26.35, 26.47, 26.82, 27.11, 27.70, 27.73, 27.78, 27.80, 28.85, 28.89, 29.08, 29.21, 31.56, 33.87, 34.03, 54.01, 54.23, 54.25, 56.56, 56.58, 56.64, 56.85, 56.91, 61.99, 68.80, 172.6, 173.10; MALDI-TOF (2577 V pulse voltage) *m*/*z* (ir) = 997.65 (100, C_57_H_98_O_12_Na, M), 999.65, (64.7 M + 2).

## 3. Results and Discussion

Considering remarkable success of methods of epoxidation using dioxirane [19, 24, 25], we decide to modify opportunely the technique which allowed for the first time the isolation of ^18^O-DDO (**1a**). In view of the fact that we need to start from a significant amount of acetone and that ^18^O-acetone is commercially available but particularly expensive [31], we chose as source of ^18^O the H_2_^18^O 95%, using it in an exchange reaction with acetone, acid-catalysed (H_2_SO_4_ 0.02 N) (1) [16]:
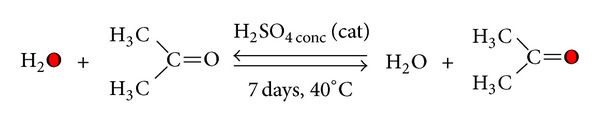
(1)


**Scheme 3:**
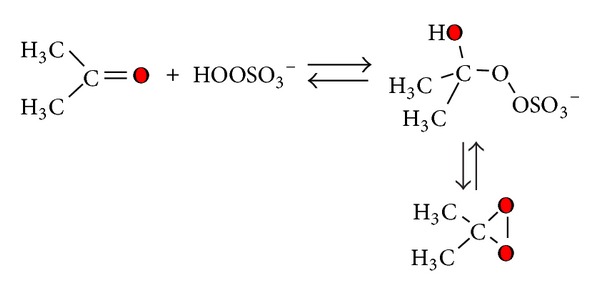


The exchange reaction was monitored by GC-MS, quantifying the two species of acetone, labelled or not, by the signal intensity of the corresponding molecular ion (*m*/*z* = 58 acetone, *m*/*z* = 60  ^18^O acetone). Finally we had a labelling for acetone equal to 69%. Dioxirane (**1a**) could be obtained by the standard procedure (Scheme 3). The only change is the amount of water, because we used the exchange mixture, without further purification.

Also in this case, it is of considerable importance that the dioxirane synthesis reaction must be perfectly buffered with NaHCO_3_, in order to minimize the loss of ^18^O-acetone labelling due to water exchange process (acid and base catalyzed). Anyway, the rate of formation of dioxirane is faster than the exchange reaction with water, thanks to the high nucleophilicity of peroxide ((2) alpha effect) [32]:
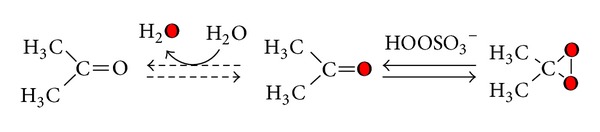
(2)


Before proceeding with the polyepoxidation of the triglyceride, in order to assess the amount of labelled of ^18^O-DDO (**1a**), it appears necessary to explore its efficacy in the labelled reaction, as both ^16^O and ^18^O oxygen atoms of the dioxirane can be transferred to *substrate *[23]. 

We chose as a target reaction the oxidation of methyl-p-tolyl-sulfide (**3**), a transformation well known for its efficiency [24]. The level of labelling on the corresponding sulfoxide (**3a**) was determined by GC-MS analysis, considering the increase of the ratio of the signal intensity M + 2/M (where M is the molecular ion), compared to the same ratio obtained by the GC-MS spectrum of a solution of methyl-p-tolyl sulfoxide standard unlabelled. In this case we obtained a labelled sulfoxide (**3a**) equal to 8% (3):
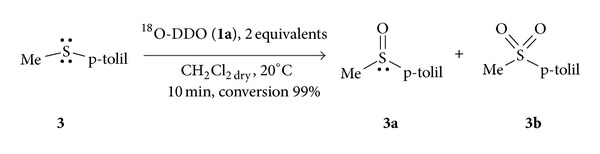
(3)


Using the data regarding the oxidation of **3** it is possible to carry out a preliminary theoretical assessment of isotopic enrichment on M + 2 signals relative to our target compound, the ^18^O-LLL_ox_ (**2a**), for which there are six successive processes of oxygen insertion. We used the Bayes formula for the determination of probability to give the percent of labelled compound: *P*_*n*,*k*_ = *n*!/*k*!(*n* − *k*)!×*p* ^*k*^ × *q* ^(*n*−*k*)*xx*^ [33] where *p* = 8/100 is the probability of the elemental event of epoxidation with ^18^O, *q* = 92/100 is the probability of the opposite elemental event, *n* = 6, and *k* is the number of ^18^O constituent the compound, product of the reaction, from 0 to 6. Based on these calculations we expected an increase of isotope peak M + 2 of our product equal to 32%, and so the theoretical possibility of obtaining a yield of 32% as product (**2a**) containing a single Atom ^18^O. The preliminary oxidation of triglyceride **2** was carried out with unlabelled DDO (**1a**) in order to optimize the reaction conditions and in particular to determine the best DDO (**1a**)/substrate ratio required for a complete substrate oxidation; afterwards, the reaction was performed using the **1a** labelled.

**Scheme 4:**
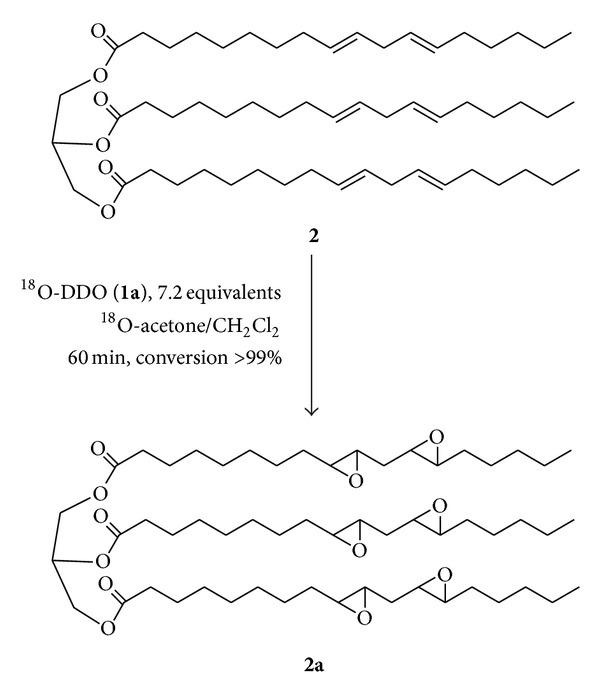


The easy oxidation procedure involved the stepwise addition (20 min) of standardized dioxirane **1a** solution to the glyceryl-three linoleate (**2**) dissolved in CH_2_Cl_2_ (20 mL) and kept at 20°C. A moderate excess of dioxirane ^18^O-DDO (**1a**) was applied in order to achieve complete substrate conversion as monitored by TLC (Scheme 4).

The GC-MS analysis of the reaction mixture showed that a significant residual of labelled acetone is still present. The precious acetone ^18^O-enriched was recovered by a careful distillation of the crude reaction.

The polyepoxide **2a** (oil) was fully characterized by ^1^H- and ^13^C-NMR, yielding spectra in complete agreement with the given structure, and the ^1^H NMR spectrum of **2a** shows *inter alias* the multiple at 2.90–3.08 (m, 12H) due to the C-*H* resonances of the epoxy ring.

**Figure 3:**
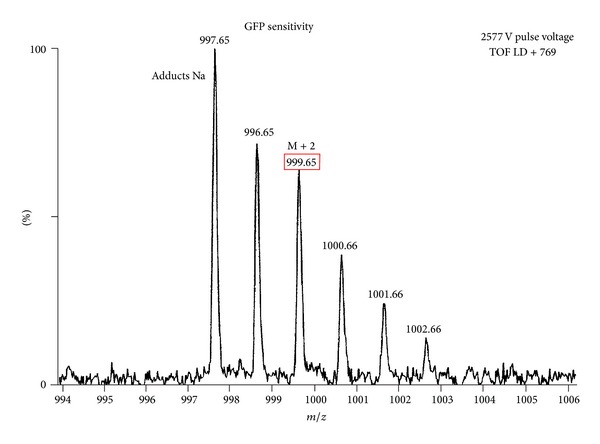
MALDI-TOF spectrum of of exa-epoxy-three linoleate glyceryl-^18^O labelled (**2a**).

In addition, the MALDI-TOF spectrum confirm the structure of the compound (noted as adducts Na + signal at *m*/*z* = 997.65) and also determine the isotopic enrichment of the M + 2 signal (*m*/*z* = 999.66). We found that it is equal to 42%, and this is in agreement with the previously calculated according to the theory of probability (Figure 3).

Data that are representative of the enrichment attainable in the direct dioxirane oxidation of different substrates are collected in Table 1. These show that, using the procedure reported herein, labelled polyepoxide **2a** can be synthesized up to 42% labelled yield. 

In summary, by the easily prepared ^18^O dimethyldioxirane (**1a**), natural products as glyceryl-three linoleate can be cleanly converted to the corresponding labelled epoxide in mild conditions. The high efficiency and selectivity of these oxidations provide highly pure products, which can be used directly in subsequent reaction steps, avoiding costly and time-consuming product purification procedures. Besides the oxidant precursor, the expensive acetone ^18^O labelled can be recovered and reused for dioxirane regeneration, thus increasing the atom economy of the process.

In conclusion, we believe that these results contribute to reinforce the notion that the application of dioxiranes efficiently provides access to useful new application not only in organic synthesis but also for the setup of new analytical methods.

## Figures and Tables

**Table 1:** Mass spectrometry data.

Products	M (*m/z*)	Unlabelled	Labelled	^ 18^O %^a^
		M + 2	M + 2	
Acetone^b^	100 (58)	1.01	69.93	69%
Methyl-p-Tolil sulfoxide (**3a**)^b^	100 (154)	5.91	13.88	8%
^ 18^O-LLL_ox_ (**2a**)^c^	100 (998)	23.23	64.71	42%
Acetone after DDO (**1a**) synthesis	100 (58)	1.01	21.02	20%

^a^The percent of ^18^O was estimated form the 100 × {[((M + 2)/M)_labelled_ – ((M + 2)/M)_unlabelled_]}.

^b^The percent of ^18^O was determined using the GC/Ms analysis.

^c^For the ^18^O-LLL_ox_ was used the MALDI-TOF analysis.
